# “Super” SERPINs—A stabilizing force against fibrinolysis in thromboinflammatory conditions

**DOI:** 10.3389/fcvm.2023.1146833

**Published:** 2023-04-19

**Authors:** Steven J. Humphreys, Claire S. Whyte, Nicola J. Mutch

**Affiliations:** Aberdeen Cardiovascular and Diabetes Centre, Institute of Medical Sciences, School of Medicine, Medical Sciences and Nutrition, University of Aberdeen, Aberdeen, United Kingdom

**Keywords:** SERPIN, thromboinflammation, plasminogen activator-inhibitor-1, α2-antiplasmin, protease nexin-1, C1-inhibitor

## Abstract

The superfamily of serine protease inhibitors (SERPINs) are a class of inhibitors that utilise a dynamic conformational change to trap and inhibit their target enzymes. Their powerful nature lends itself well to regulation of complex physiological enzymatic cascades, such as the haemostatic, inflammatory and complement pathways. The SERPINs α2-antiplasmin, plasminogen-activator inhibitor-1, plasminogen-activator inhibitor-2, protease nexin-1, and C1-inhibitor play crucial inhibitory roles in regulation of the fibrinolytic system and inflammation. Elevated levels of these SERPINs are associated with increased risk of thrombotic complications, obesity, type 2 diabetes, and hypertension. Conversely, deficiencies of these SERPINs have been linked to hyperfibrinolysis with bleeding and angioedema. In recent years SERPINs have been implicated in the modulation of the immune response and various thromboinflammatory conditions, such as sepsis and COVID-19. Here, we highlight the current understanding of the physiological role of SERPINs in haemostasis and inflammatory disease progression, with emphasis on the fibrinolytic pathway, and how this becomes dysregulated during disease. Finally, we consider the role of these SERPINs as potential biomarkers of disease progression and therapeutic targets for thromboinflammatory diseases.

## Introduction

**Figure 1:**
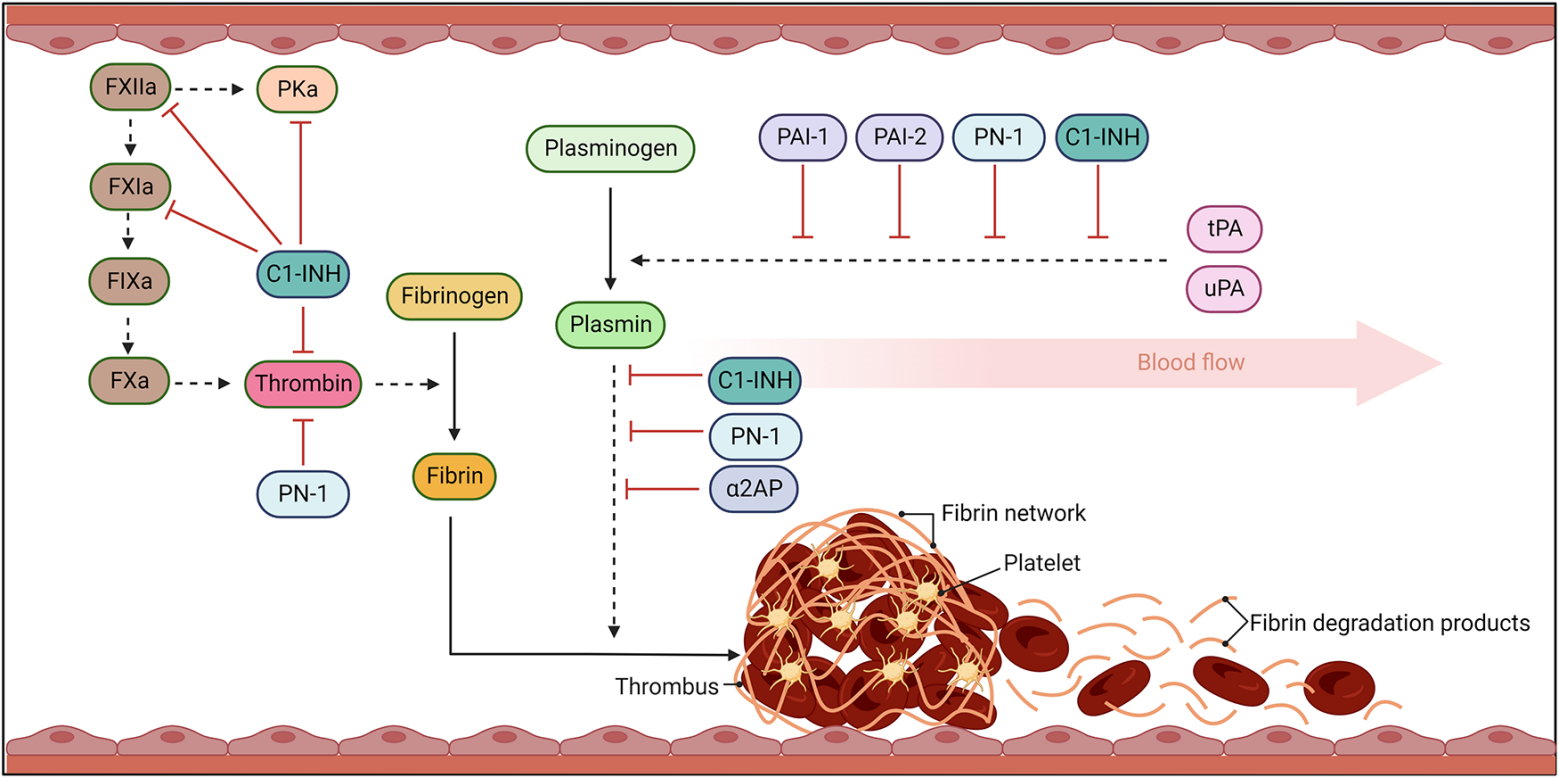
The fibrinolytic response. Coagulation results in thrombin cleavage of fibrinogen into fibrin. Fibrin threads become crosslinked, trapping platelets and blood cells, stabilising the clot. Once bleeding has been controlled and damage to vessel repaired, the fibrinolytic pathway acts to dissolve the fibrin network. Fibrinolysis occurs when plasminogen activators, urokinase- (uPA) and tissue-type- (tPA), cleave plasminogen to plasmin. Plasmin then degrades fibrin into fibrin degradation products (FDP) which can be efficiently cleared from the circulation. Serine protease inhibitors (SERPINs) act to inhibit the activity of these serine proteases to avoid uncontrolled activity that can lead to bleeding complications and dysregulated activity. Solid arrows signal catalytic conversion of proteins and enzymes from inactive to active states, while dashed arrows signal proteolytic activity. Blunt red arrows denote the specific inhibition of enzymes by different SERPINs.

Serine proteases are powerful proteolytic enzymes that function in crucial pathways such as coagulation, fibrinolysis, inflammation, and the complement system (1–3). The general mechanism of action of these proteases is cleavage of peptide bonds, thereby converting proteins from inactive zymogens to active enzymes. The prolific nature of these serine proteases requires exquisite regulation which is the task of the superfamily of serine protease inhibitors (SERPINs). In most SERPINs the amino acid sequence in the reactive center loop (RCL) mimics that of the enzymes target protease and upon cleavage the serine protease is dynamically “trapped” in an irreversible 1:1 complex (4–6), leading to the classification of SERPINs as suicide inhibitors. The detailed structure and mechanism of SERPINs has recently been elegantly reviewed and will not be described in depth herein (7). SERPINS with a role in haemostasis include antithrombin, heparin cofactor II, protein-Z-dependent protease inhibitor, and α1-antitrypsin. This review focuses on SERPINs with a role in fibrinolysis (8, 9). Although there is some evidence that α1-antitrypsin participates in fibrinolysis its primary role is regulation of neutrophil-derived proteases (10–14). SERPINs like α2-antiplasmin (α2AP), plasminogen-activator inhibitor-1 and -2 (PAI-1 and -2), protease nexin-1 (PN-1), and C1-inhibitor (C1-INH) play crucial functions in the fibrinolytic system, by inhibiting plasmin, tissue-type plasminogen activator (tPA), and urokinase-type plasminogen activator (uPA) (Figure 1) (9, 15). Elevated levels of several SERPINs have been associated with a plethora of disease states including type 2 diabetes, obesity, metabolic syndrome, and hypertension. They have been identified as prognostic markers in several cancers including, renal, breast, lung, head and neck, colorectal, and ovarian cancers (16–19) and have been implicated in cancer progression and metastasis. These areas fall out with the scope of this review and have been described in detail elsewhere (20, 21). In this review we will focus on the biological function of the SERPINs that modulate fibrinolysis, how this is perturbed during thromboinflammatory diseases, as well as highlight existing and novel aspects to exploit for novel therapeutics.

## α2-Antiplasmin—“the alpha SERPIN”

**Figure 2:**
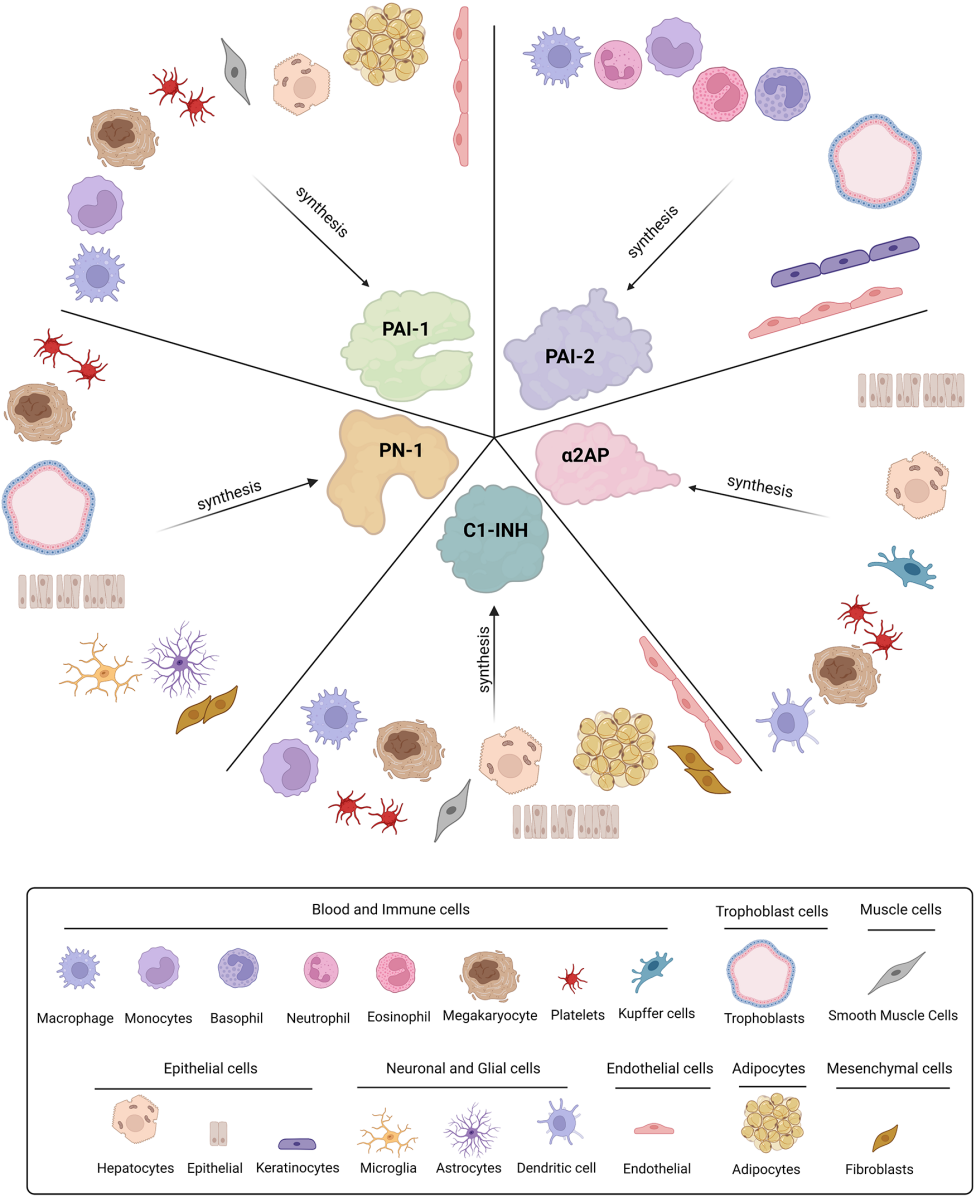
Cellular origins of SERPINs involved in fibrinolysis. Plasminogen activator-1 and -2 (PAI-1 and PAI-2), Protease nexin-1 (PN-1), alpha2-antiplasmin (α2AP), and C1-inhibitor (C1-INH) are all synthesised by various cell types throughout the body.

α2AP is the principal inhibitor of plasmin *in vivo* (22). It is an unusual SERPIN as its extensive C-terminal lysine-rich tail initially docks to the kringles of plasmin forming a non-covalent complex (23). Following plasmin cleavage of the RCL a stable covalent 1:1 plasmin-antiplasmin (PAP) complex is formed. The main plasma pool of α2AP is secreted by hepatocytes (Figure 2) (24) and circulates at 70 µg/ml, over 1,000-fold higher than PAI-1 (25). Despite being the dominant fibrinolytic SERPIN in plasma its level is surprisingly still lower than the zymogen concentration of its target enzyme, plasminogen (200 µg/ml) (26). However, the inhibitor is covalently cross-linked to fibrin through the action of the transglutaminase, activated factor XIIIa, during thrombus formation thereby localising it at the site of action and enhancing its efficacy (27, 28). Our laboratory has previously shown that cross-linking of α2AP into a thrombus is dependent on flow or shear stress (29) and others have demonstrated that compaction of clots is sufficient to promote the crosslinking reaction (30). Interestingly, despite its plethora of other substrates, the antifibrinolytic action of factor XIIIa is exclusively accounted for by cross-linking of α2AP (31) illustrating the “alpha” role of this SERPIN in modulating fibrinolysis. α2AP exists in several forms within the circulation (32). The secreted form, Met-α2AP, is cleaved by α2AP cleaving enzyme (APCE) to form Asn-α2AP (33) which cross-links thirteen times faster to fibrin, due to exposure of glutamine 2 in this truncated form (32). The C-terminus is post-translationally modified by an as yet unidentified enzyme in the circulation with downstream impact on the interaction with plasmin(ogen) (32).

Dysregulation of α2AP disrupts the fibrinolytic balance leading to a risk of thrombosis or bleeding (34, 35). Congenital α2AP deficiencies, in which the mutation perturbs protein levels and/or function are associated with excessive or delayed bleeding following trauma and/or spontaneous rebleeding (36, 37). An acquired deficiency in α2AP can occur due to infusion of thrombolytic agents and is commonly associated with severe liver disease and acute leukaemia (36, 37). In contrast, elevated levels of α2AP are linked with diseases such as atherosclerosis thereby provoking the risk of ischemic stroke (IS) and myocardial infarction (MI) (38, 39). Experimental models of IS indicate that elevated levels of α2AP lead to a reduction in thrombus dissolution and enhanced necrosis, while neutralisation of this SERPIN during thrombolytic therapy significantly improves outcomes (40). Increased levels of α2AP are directly linked to adverse outcomes in terms of ischemic brain injury, swelling, microvascular thrombosis and mortality following cerebral thromboembolism (41). Several avenues have been pursued to neutralise the activity of α2AP, including microplasmin and plasmin infusions, neutralising antibodies, and synthetic peptides (42–44). In a mouse model of IS infusion of plasmin or immunoneutralization was shown to temporarily lower systemic α2AP levels for around 24 h (43).

Significantly elevated levels of circulating α2AP have been identified as a key component in the pathophysiology of pulmonary arterial hypertension (PAH), contributing to poorer outcomes and higher mortality (45, 46). Reduced interactions between prekallikrein, factor XI (FXI) or α2AP, with small high-density lipoprotein subclasses correlate with higher mortality in PAH patients (47). A significant relationship has also been observed between α2AP and obstructive sleep apnoea (OSA), cardiomyopathy, congestive heart failure, and atrial fibrillation (48). Interestingly, increased levels of both α2AP and PAI-1 correlate with augmented prothrombotic activity in patients with OSA (49). These data suggest that inhibition of these SERPINs may be potential therapeutic option to down-regulate thrombotic complications in OSA. Interestingly, a significant decrease in α2AP activity has been observed in arterial hypertension, reflecting a shift to a hyperfibrinolytic state (50). The implication of these findings is not year clear and requires further exploration.

Therapeutic targeting of α2AP is an attractive strategy which could mitigate the bleeding risk associated with conventional anticoagulant and thrombolytic therapy. A phase 1 clinical trial in patients with acute lower extremity arterial or bypass occlusion indicated that catheter-delivered plasmin at high doses significantly reduced systemic α2AP by 39% resulting in >50% clearance of the thrombus in 79% of patients (51). This interesting study indicates that consumption of endogenous α2AP is a safe and effective way of promoting thrombolysis *in vivo*. A novel monoclonal antibody that inactivates α2AP, TS23 (42, 52), has been used in phase 1 clinical trials in healthy individuals, but outcomes are not yet available. A randomised phase 2 clinical trial was started earlier this year to evaluate the efficacy of TS23 in treating acute pulmonary embolism (53), with results expected in 2024. These developments are promising and signify considerable interest in targeting this SERPIN for a variety of thrombotic and cardiovascular conditions and one would hope that new drugs and compounds directed toward α2AP may be on the horizon in the not-too-distant future.

## Plasminogen activator inhibitor-1—“the premier PAI”

PAI-1 is a 45 kDa glycoprotein and the “premier” inhibitor of plasminogen activators (PAs) (54). PAI-1 is highly expressed in many tissues, including placenta, gallbladder, liver, lung, and kidney as well as various cell-types such as platelets, syncytiotrophoblasts, adipocytes, smooth muscle cells (SMCs), endothelial cells, and fibroblasts (Figure 2) (55–59). Levels of PAI-1 in plasma are relativity low (20 ng/ml) (60) with the main circulating pool found within the α-granules of platelets (61). PAI-1 is expressed by megakaryocytes, the precursor cells of platelets (62, 63) and is packaged into α-granules during biogenesis. However, mRNA can be found within platelets where the inhibitor is synthesised to a limited degree in its active form (62). Unlike other SERPINs, PAI-1 can spontaneously transition from its unstable active form (half-life ∼1 h) (64), to a more thermodynamically stable latent form, extending the half-life to ∼2–4 h (65). Plasma PAI-1 levels are known to oscillate in a circadian rhythm, peaking in the morning (66, 67) thereby reducing fibrinolytic potential (68). This diurnal variation in PAI-1 has been postulated to explain the morning peak in adverse cardiovascular events (69, 70). Circulating levels of PAI-1 are influenced by the 4G/5G genetic polymorphism in the promoter region (71). Elevated levels of PAI-1 in the morning are most apparent in homozygotes for 4G and intermediate for heterozygotes (72, 73).

Deficiency in PAI-1 is associated with hyperfibrinolysis, which can be either congenital or acquired. A complete PAI-1 deficiency is characterised by a mild-moderate bleeding diathesis most commonly associated with trauma or surgery (74). Despite equal prevalence in males and females, PAI-1 deficiency is usually diagnosed earlier in females due to menorrhagia and postpartum haemorrhage (75). Acquired deficiency in PAI-1 is a common consequence of cirrhosis of the liver and observed clinically as increased levels of circulating D-dimers and fibrin degradation products (FDPs) (76–78). Conversely, elevated PAI-1 incites a hypofibrinolytic state, which has been linked to increased risk of venous and arterial thrombosis (79, 80).

Elevated levels of PAI-1 have been correlated with IS (81–83). Similarly increased levels of inflammatory markers and fibrinolytic inhibitors, including PAI-1, were recorded in intracranial large artery atherosclerosis (ILA), a major cause of IS (81, 84). Importantly, PAI-1 and C-reactive protein could predict ILA progression independent of other biomarkers assessed; these data highlight the unique relationship between pro-inflammatory and hypofibrinolytic states (81). Elevated plasma levels of TNF-α, PAI-1, and tPA, markers of immuno-inflammatory activation and endothelial dysfunction, correlate with stroke diagnosis (85). The “sterile” thromboinflammatory state observed in stroke patients is observed in other conditions such as venous thrombosis, diabetes and autoimmune diseases (86). A retrospective observational clinical trial is currently investigating differences in factor VIII, tPA, and PAI-1 as biomarkers for the occurrence of intracranial haemorrhage in patients with acute IS, pre- and post-treatment with alteplase (87). A population-based case-control study demonstrated that elevated levels of PAI-1 are predictive of venous thrombosis (88). Interestingly, a recent study investigating deep vein thrombosis (DVT) in patients following total hip arthroplasty, found that PAI-1 expression was a postoperative biomarker (89). Similarly, PAI-1, fibrinogen, and D-dimers were predictive of postoperative DVT following surgery for lower limb trauma (90). Collectively these studies highlight the prognostic power of PAI-1 in a variety of thromboinflammatory conditions, however, this would require further work to establish and standardise normal ranges.

Attenuated fibrinolysis is a significant factor in obesity-associated thrombosis, attributed to an increase in PAI-1 levels (91–93). Elevated gene expression and plasma tPA are also observed in obesity which is suggested to be a compensatory mechanism to overcome PAI-1, despite this, hypofibrinolysis persists (94, 95). Adipocytes are proposed as the principal source of PAI-1 with enhanced synthesis primarily driven by the proinflammatory cytokines TNF-α and TGF-β1 (93, 96, 97). Interestingly, in healthy individuals there was no arterio-venous differences in PAI-1 antigen or activity across an adipose tissue bed (98). An interesting study on glycosylation of PAI-1 suggested that in lean individuals the primary source of PAI-1 is platelets while in obese subjects the glycosylation pattern of plasma PAI-1 mimicked that of adipose tissue, suggesting these cells contribute to circulating levels (99). Recently hepatocytes have been described as an alternative source of increased PAI-1 and tPA in obesity due to alterations in the hepatic PAI-1/tPA gene regulatory pathways (95). Further research into the various cellular sources of plasma PAI-1 could be fruitful for future targeting of this SERPIN in various cardiometabolic diseases.

The most frequent cause of MI is rupture of atherosclerotic plaque, leading to occlusive thrombus formation. Many cells that express PAI-1, such as platelets, macrophages, and SMCs reside within the plaque milieu (100–102). Atherosclerotic progression is associated with platelet activation and PAI-1 secretion thereby promoting thrombus resistance to lysis (103–106). Recurrence of MI has also been linked to increased plasma levels of PAI-1 (107, 108). It was suggested that this causality may arise due to the multifaceted role of PAI-1 in aging, fibrinolysis, and endothelial injury (109, 110), with elevated levels provoking a prothrombotic and profibrotic state, thereby impairing clot breakdown and driving endothelial dysfunction (111). The onset of acute MI follows diurnal oscillations, with most cases occurring in the morning between 6:00 am and noon (112). Misalignment between the internal and external clock, for instance during daylight saving time, have been associated with increased risk of MI (113). A recent study on acute MI determined a correlation between tPA and PAI-1 levels with highest levels recorded in the morning (114), in agreement with earlier studies (66–68, 70). However, treatment with the anti-platelet drug clopidogrel gave rise to elevated levels of PAI-1 in those acute MI patients resistant to clopidogrel, but not clopidogrel-sensitive patients (114). These interesting data suggest that increased evening PAI-1 may relate to platelet activation and underscore the diurnal changes that occur both naturally in this SERPIN and in response to drugs. Several clinical trials are currently investigating PAI-1 in different thromboembolic conditions. Eplerenone, a selective aldosterone receptor antagonist, was shown to attenuate the morbidity and mortality of patients with acute MI (115), and is being analysed to reduce PAI-1 levels in aldosterone-induced MI in a randomised control study (116). A first-in-class orally available PAI-1 inhibitor for the treatment of fibrosis and fibroproliferative disorders is currently in phase 1 clinical trials (117). Metformin, a type-2 diabetes treatment, is currently being investigated in a phase 4 non-randomised trial for its use in PAI-1 deficient individuals to prevent/stabilise cardiac fibrosis (118).

During the COVID-19 pandemic thromboembolic complications were quickly recognised as a consequence of severe disease (119, 120). Reduced fibrinolytic capacity, observed as prolonged clot lysis, increased clot strength, and elevated platelet counts, were observed in 70% of COVID-19 patients (121). This has been attributed to variations in fibrinolytic factors such as PAI-1 (>2-fold), tPA (>1.6-fold), and thrombin activatable fibrinolysis inhibitor (TAFI) (>1.7-fold) in critically ill COVID-19 patients (122). Dysregulation of haemostasis in patients with severe COVID-19 is linked to an aberrant hyperinflammatory state triggered by the cytokine storm (123). We demonstrated elevated PAI-1 in a cohort of hospitalised COVID-19 patients that supressed plasmin generation and tPA-mediated lysis thereby driving the suboptimal fibrinolytic response (124). Interestingly, a direct association between PAI-1 and hypofibrinolysis was also observed during the SARS-CoV outbreak of 2002 (125). A double-blind randomised phase 2 clinical trial of a novel PAI-1 inhibitor (TM5614) to evaluate its efficacy in treating severe COVID-19 (126) has unfortunately been suspended due to challenges in patient recruitment and drug manufacturing issues. Similarly, a case-control prospective clinical trial to assess the predictive power of PAI-1 in the development of severe COVID-19 was initiated but has not been updated suggesting issues with recruitment (127).

These studies and observations indicate that PAI-1 is a powerful player in defining the physiological and pathophysiological response. A diverse portfolio of PAI-1 inhibitors, including antibodies, nanobodies, antibody fragments, and peptides, have been reported in the literature but we are yet to see a drug traverse the pipeline for clinical use (128–130).

## Plasminogen activator inhibitor-2—“the incognito PAI”

PAI-2 is expressed in multiple cells, such as monocytes, granulocytes, trophoblasts, epithelial and endothelial cells (Figure 2) (131–133). Elevated PAI-2 is observed in pregnancy and is related to increased cellular expression by the trophoblastic epithelium of the placenta (134). PAI-2 lacks a signal peptide for release (135), and therefore its function is presumed to be intracellular, as reflected by the negligible levels in plasma. Nonetheless, this “incognito” SERPIN has the capacity to inhibit many extracellular proteases, despite reduced efficacy toward tPA and uPA compared to PAI-1 (136, 137). Studies related to PAI-2 in pathophysiological states are limited, however there are reports to suggest that PAI-2 can modulate venous thrombus (138), coronary artery disease (139, 140), and cerebral artery occlusion (83). Upregulation of PAI-2 has also been observed in patients with acute respiratory distress syndrome (ARDS) (141). Interestingly, PAI-2 expression is markedly increased after exposure to inflammatory stimuli such as TNF-α, suggesting a role for this SERPIN in inflammation (131, 132) as well as other processes such as apoptosis (142), infection (143) and cancer (144).

An instrumental study into the role of PAI-2 in fibrinolysis demonstrated that venous thrombi from PAI-2 deficient mice harboured 12-fold higher levels of active uPA (138). There was also pronounced improvement in thrombus resolution in both PAI-1 and PAI-2 deficient mice; this is likely linked to the overexpression of uPA and the observed infiltration of inflammatory cells, such as neutrophils and macrophages (138, 145, 146). Interestingly, enhanced monocyte-derived uPA activity (150-fold) has been associated with reductions in PAI-1 and PAI-2, 1.6- and 2.1-fold respectively, and more efficient dissolution of thrombi (146–148). In contrast, formation of thrombi was only impaired by PAI-1 deficiency (138), indicating a complex interplay of these two SERPINs in venous thrombus formation and resolution.

PAI-1 and PAI-2 have been investigated in IS, in a model of middle cerebral artery occlusion (MCAO) (83). They found that 24 h post-stroke, levels of PAI-2 and PAI-1 mRNA were significantly overexpressed, 19- and 237-fold, respectively. PAI-1 deficient mice had a 31% decrease in brain infarct volume 24 h post-MCAO, however this was not observed in PAI-2 deficient mice, suggesting this SERPIN has limited impact on secondary brain damage post-stroke (83). The role of PAI-1 in brain injury is well established but given the limited studies on PAI-2 the significance of this SERPIN in this setting remains unclear (109, 149, 150).

Clearly, we currently lack an understanding of the relevance of PAI-2 in both the intracellular and extracellular space. The distinct elevation of plasma PAI-2 in pregnancy and the relationship with inflammation demands further attention and underscores are limited knowledge. PAI-2 has been presumed to function intracellularly yet the limited studies available indicate that it can “escape” to the circulation which may be promoted under certain pathophysiological conditions and/or environmental settings.

## Protease nexin-1—“the binary SERPIN”

PN-1 was originally identified in glial cells in the 1970s (151) and has subsequently been found in a variety of cells including epithelial, fibroblasts, glial cells, and trophoblasts (Figure 2) (152, 153). Within the SERPIN superfamily PN-1 is the phylogenetically closest relative to PAI-1, sharing both structural and functional homologies (154, 155). PN-1 levels are negligible in plasma but, like its phylogenetic cousin PAI-1, it is found at high concentrations within platelet α-granules (156). Activated platelets secrete PN-1 in response to agonist stimulation which attenuates plasmin generation and activity (156). PN-1 demonstrates “binary” function as it targets the primary serine proteases of the coagulation and fibrinolytic cascade, thrombin and plasmin, as well as other enzymes within these pathways including factor XI (FXI), tPA and uPA (156–158).

In the last two decades the focus on PN-1 has intensified with various links to haemophilia (159) and fibrosis (160) reported. Cardiac fibrosis is defined as an excessive accumulation of extracellular matrix (ECM) proteins (161) promoting myocardial stiffness and altered systolic function (162). Elevated PN-1 was recorded both *in vivo* and *in vitro* in mouse models of fibrosis (163), with mRNA expression increased 5.5- and 1.9-fold in the plasma and myocardium, respectively. This increase is hypothesised to be attributed to PN-1 inhibition of uPA in the extracellular space (164). Aspirin attenuates PN-1 levels in cardiac fibrosis due to repressed phosphorylation of Erk1/2-PN-1 and blockage of the MAPK-Erk1/2-PN-1 pathway in cardiac fibroblasts, suggesting utility as a drug target or marker of disease progression (163, 165). There are currently no drugs with antifibrotic activity approved for clinical use (166), although PAI-1 has been highlighted (117, 118). Given the close phylogenetic connection between these SERPINs further work into the role of PN-1 in this setting could be of value.

PN-1 inhibits thrombin generation and activity *in vitro*, more efficiently than other SERPINs, with an inhibitory constant (Ki) of 1.41 × 10^−6^ M^−1^s^−1^ (167) compared to Ki of 1.08 × 10^−4^ M^−1^s^−1^ for antithrombin (168). In PN-1 deficient mice thrombus formation was augmented, as characterised both in *ex vivo* collagen-induced flow models and *in vivo* FeCl_3_ induced injury (169). Neutralising antibodies to PN-1 enhance thrombin activity in mild to moderate haemophilia A and mild haemophilia B patients highlighting its potential as a treatment for haemophilia activity (159, 170). These studies reveal that the role of PN-1 in thrombin inhibition could be exploited as a future pharmacological intervention. Nonetheless, the capacity of this “binary SERPIN” to also inhibit plasmin and uPA *in vivo* must be teased out before development of therapeutic strategies.

## C1-inhibtor—“the bountiful SERPIN”

C1-INH is the most abundant protease inhibitor in plasma circulating at a massive 250 µg/ml (171), perhaps reflecting the diversity and number of protease targets. It was conventionally considered an inhibitor of the complement pathway (C1r and C1s) and the contact system (FXIIa, FXIa, and kallikrein), however, it has also been shown to target enzymes in the fibrinolytic pathway, including plasmin, tPA, and uPA (15, 172). C1-INH is primarily produced by the liver, but is expressed in other cell types, such as monocytes/macrophages, endothelial cells, SMCs and fibroblasts (Figure 2) (173–176). Platelet α-granules also harbour C1-INH although this accounts for only 0.08% of the total circulating pool (177, 178).

A deficiency or impaired function of C1-INH is associated with angioedema, both hereditary (HAE) and acquired (179, 180). Acute attacks in HAE patients (C1-INH-HAE) are characterised by swelling of deep tissue and mucosa, as a consequence of local vascular leakage and an increase in bradykinin (181). Interestingly, the most common sites of swelling in these patients are in areas of high fibrinolytic activity, such as the lips, mouth, tongue, eyelids and genitalia (182, 183). A recent study investigating the risk of comorbidities in HAE found increased risk of hypertension, arterial and venous embolism, in line with C1-INH's multifunctional role in various pathways (184, 185). Antifibrinolytic drugs, such as tranexamic acid can reduce the severity of HAE attacks (186, 187), however the efficacy of this treatment is exceptionally variable (188, 189). There are many studies, both interventional and observational, investigating C1-INH deficiency, but the majority focus on the link with angioedema (190–193). Interestingly, angioedema is a common side-effect of thrombolytic therapies (194, 195) suggesting further linkage between these pathways, however, C1-INH function in the fibrinolytic pathway has largely been overlooked and understudied (171, 196). To add to the complexity while C1-INH directly inhibits both plasmin and tPA, it can itself be inactivated by plasmin cleavage (171). Additionally, there have now been several studies to indicate an increase in various prothrombotic plasma proteins, including factor XIIa, prothrombin, D-dimer and thrombin generation in C1-INH deficient patients (181, 197–199). Replacement therapies with human C1-INH reduce circulating levels of these markers thus lowering thrombotic risk (200, 201). C1-INH deficient mice also show increased activation of the coagulation pathway and venous thrombosis (202). Interestingly, in the general population low levels of C1-INH correlate with an increased risk of venous thrombosis (203).

C1-INH has been investigated in COVID-19 disease, partly due to its similarities in disease progression as HAE, including immune activation driving inflammation, endothelial dysfunction, and altered fibrinolysis (204, 205). Elevated C1-INH was identified in patients with severe, but not non-severe COVID-19 (206, 207). Current randomised clinical trials are studying C1-INH in relation to COVID-19 to reduce disease progression (208) and its use as a co-therapy with icatibant, a competitive antagonist to bradykinin, on the pulmonary manifestations of COVID-19 disease (209).

C1-INH is critical in the inhibition of multiple enzymes/factors in the contact, complement, and fibrinolytic systems (172). Research in this field is divided on whether there are thrombotic risks associated with C1-INH deficiency, accentuating an opportunity to bridge gaps in our knowledge. The “bountiful” array of protease targets of this SERPIN in the inflammatory, haemostatic and complement pathways has directed attention toward its value as a therapeutic and predictive biomarker, with ongoing clinical studies highlighting its potential in various thromboinflammatory conditions.

## Concluding remarks

SERPINs play multi-functional roles targeting serine proteases in the complement, coagulation and fibrinolytic pathways, orchestrating a fine balance between promotion and inhibition of these pathways. In the last few decades, many studies have helped decipher the behaviour of α2AP, PAI-1, PAI-2, PN-1, and C1-INH in thrombotic and inflammatory diseases. Primarily these SERPINs have been suggested to have predictive powers, although their precise role in different disease settings has not been fully disentangled and additional studies would be necessary to standardise normal ranges. Nevertheless, elevated levels of these SERPINs are related to the incidence, severity, and prognosis of various thromboembolic diseases, including ischemic stroke, myocardial infarction, along with pathogenic infections, such as COVID-19 and sepsis, and inflammation, such as angioedema. Evidently, the power and regulatory capacity of PN-1 and PAI-2 are understudied thereby currently precluding their viability as therapeutics, but yet leave the door open to future research in the field. However, there is currently significant interest in targeting α2AP, PAI-1, and C1-INH in a variety of thromboembolic and inflammatory conditions. Understanding the complex interplay of SERPINs in regulating the fibrinolytic response is imperative to define their critical role in maintaining haemostasis and will provide clues as to the intertwined relationship between the fibrinolytic, inflammatory and complement systems. Despite the current state of knowledge and rational for various SERPINs as therapeutics, there are currently an exceptionally limited repertoire of drugs with regulatory approval to target these inhibitors. With the current interest in thromboinflammation it is a prime opportunity to propel these “super” SERPINs into the limelight to understand their biological function and utility as therapeutic targets.
